# Reply: Strengths and Limitations of the Renal Locoregional Perfusion Platform

**DOI:** 10.1016/j.jacbts.2026.101591

**Published:** 2026-06-22

**Authors:** Maximilian Y. Emmert, Johannes Holzmeister, Volkmar Falk, Josef El Andari

**Affiliations:** a Department of Cardiothoracic and Vascular Surgery, Deutsches Herzzentrum der Charite (DHZC), Berlin, Germany; b Charité-Universitätsmedizin Berlin, Corporate Member of Freie Universität Berlin and Humboldt-Universität zu Berlin, Berlin, Germany; c Institute for Regenerative Medicine (IREM), University of Zurich, Schlieren, Switzerland; d DZHK (German Centre for Cardiovascular Research), Partner Site Berlin, Berlin, Germany; e DiNAQOR AG, Schlieren, Switzerland; f DiNATEQ AG, Schlieren, Switzerland; g Department of Health Sciences and Technology, ETH Zurich, Zurich, Switzerland

We thank Dr Luque-Rincon for the supportive comments and his important questions.^1^ We are aware that our loco-regional perfusion (LRP) concept comes with some complexity, as it requires profound cardiovascular and technical expertise in organ-specific hemodynamics, extracorporeal perfusion (ie, extracorporeal membrane oxygenation), and catheter-based technologies. Clinically, we consider the LRP concept a prime example of an advanced cardiovascular hybrid approach for high-precision therapeutics delivery. As such, and as for any innovative technology, systematic implementation with a certain learning curve is required to enable broad clinical adoption.

However, because gene therapies are usually performed in large tertiary or academic centers, the mandatory infrastructure and multidisciplinary experts to perform LRP procedures are mostly already available. Such hospitals will therefore play a pivotal role in the clinical implementation process and serve as “centers of excellence” to provide advice and training on LRP procedures, thereby boosting broad clinical adoption.

To that end, this is why in our study we put particular emphasis on clinical translatability, and accordingly performed all our preclinical studies in a highly translational set-up, mimicking real-world scenarios. Notably, and unlike other more invasive ex vivo organ-perfusion protocols, our LRP concept is the first to employ a fully percutaneous in vivo perfusion approach, which substantially enhances its translational readiness.

We used custom-made catheters that were designed to meet the necessary kidney-specific key performance parameters and anatomical boundary conditions. Notably, all other components are clinically used materials. To ensure broad access to the LRP platform, we would foresee commercial availability of LRP-specific catheters in the future.

Similar to any other type of extracorporeal circuit (eg, cardiopulmonary-bypass [CPB]), active LRP-circuit management is necessary. These tasks are usually performed by highly skilled perfusionists. These specialists are available in most tertiary hospitals and an integral part of any team performing cardiac surgical procedures. As such, the rapid development of LRP-specific perfusion regimens and guidelines (analogous to CPB guidelines)^2^ by a group of experts that is familiar with all challenges of extracorporeal perfusion systems is very realistic. Beyond that, we are currently developing integrated LRP consoles to further enhance user-friendliness of LRP-circuit management.

The understanding of hemodynamic tolerance in patients with impaired renal compliance or chronic kidney disease is a critical translational consideration. We recognize that high-flow recirculation needs to be carefully individualized in patients with compromised vascular beds, ultimately prompting the need for the preprocedural consideration of disease- and patient-specific hemodynamic-parameters. Here, our LRP-circuit surveillance strategy, which allows for continuous circuit management including flow, pressure, reservoir levels, and vasodilator doses, represents an important basis for efficient organ preservation.

To overcome the autofluorescence challenge, our findings were validated using objective molecular assays, including enzyme-linked immunosorbent assay to quantify specific protein expression in the kidney. To identify specific cell types, we obtained additional unpublished data using RNAscope probes designed specifically to detect the adeneo-associated virus genome within the nuclei of tubular cells and other renal compartments. This avoids the limitations of autofluorescence, as the signal is observed as distinct intranuclear puncta rather than general green background.

We concur with Dr Luque-Rincon that exploring simplified renal perfusion approaches represents a valuable next step, and that our novel LRP concept may serve as a benchmark for future translational innovations in organ-targeted therapeutics, which also has the potential for a number of applications beyond the kidney.
